# DNA methylation of *FKBP5* in South African women: associations with obesity and insulin resistance

**DOI:** 10.1186/s13148-020-00932-3

**Published:** 2020-09-21

**Authors:** Tarryn Willmer, Julia H. Goedecke, Stephanie Dias, Johan Louw, Carmen Pheiffer

**Affiliations:** 1 Biomedical Research and Innovation Platform, South African Medical Research Council, Tygerberg, 7505 South Africa; 2 Division of Medical Physiology, Faculty of Health Sciences, Stellenbosch University, Tygerberg, 7505 South Africa; 3 Non-Communicable Diseases Research Unit, South African Medical Research Council, Tygerberg, 7505 South Africa; 4 Division of Exercise Science and Sports Medicine, Department of Human Biology, University of Cape Town, Boundary Road, Newlands, 7700 South Africa; 5 Department of Biochemistry and Microbiology, University of Zululand, Kwa-Dlangezwa, 3886 South Africa

**Keywords:** Obesity, Insulin resistance, Glucocorticoid receptor, FKBP5, DNA methylation, Adipose tissue

## Abstract

**Background:**

Disruption of the hypothalamic–pituitary–adrenal (HPA) axis, a neuroendocrine system associated with the stress response, has been hypothesized to contribute to obesity development. This may be mediated through epigenetic modulation of HPA axis-regulatory genes in response to metabolic stressors. The aim of this study was to investigate adipose tissue depot-specific DNA methylation differences in the glucocorticoid receptor (*GR*) and its co-chaperone, FK506-binding protein 51 kDa (*FKBP5*), both key modulators of the HPA axis.

**Methods:**

Abdominal subcutaneous adipose tissue (ASAT) and gluteal subcutaneous adipose tissue (GSAT) biopsies were obtained from a sample of 27 obese and 27 normal weight urban-dwelling South African women. DNA methylation and gene expression were measured by pyrosequencing and quantitative real-time PCR, respectively. Spearman’s correlation coefficients, orthogonal partial least-squares discriminant analysis and multivariable linear regression were performed to evaluate the associations between DNA methylation, messenger RNA (mRNA) expression and key indices of obesity and metabolic dysfunction.

**Results:**

Two CpG dinucleotides within intron 7 of *FKBP5* were hypermethylated in both ASAT and GSAT in obese compared to normal weight women, while no differences in *GR* methylation were observed. Higher percentage methylation of the two *FKBP5* CpG sites correlated with adiposity (body mass index and waist circumference), insulin resistance (homeostasis model for insulin resistance, fasting insulin and plasma adipokines) and systemic inflammation (c-reactive protein) in both adipose depots. *GR* and *FKBP5* mRNA levels were lower in GSAT, but not ASAT, of obese compared to normal weight women. Moreover, *FKBP5* mRNA levels were inversely correlated with DNA methylation and positively associated with adiposity, metabolic and inflammatory parameters.

**Conclusions:**

These findings associate dysregulated *FKBP5* methylation and mRNA expression with obesity and insulin resistance in South African women. Additional studies are required to assess the longitudinal association of *FKBP5* with obesity and associated co-morbidities in large population-based samples.

**Graphical abstract:**

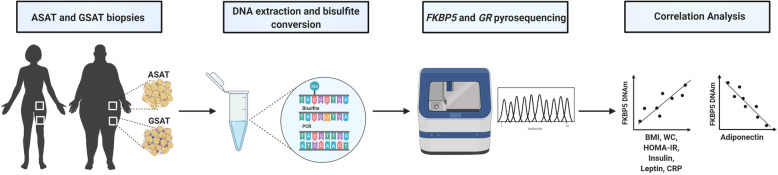

## Background

The pathophysiology of obesity involves a complex interplay between genetic and environmental influences [1]. The interaction between these factors in disease development may be mediated through dysregulation of the hypothalamic–pituitary–adrenal (HPA) axis, a neuroendocrine system that plays a critical role in maintaining metabolic homeostasis in response to metabolic stressors, such as chronically high ambient levels of stress, reduced physical activity and consumption of high caloric foods that are readily available in a Western society [2]. The activity of the HPA axis is regulated by glucocorticoids, which convey their signals through the glucocorticoid receptor (GR), a transcriptional regulator [3]. The sensitivity of cells to glucocorticoid action is tightly regulated in part, through the interaction of GR with the co-chaperone, FK506-binding protein 51 kDa (FKBP5), which negatively regulates GR signaling by inhibiting its binding to glucocorticoids and impeding translocation to the nucleus [4]. In turn, *FKBP5* expression is transcriptionally activated by GR, which binds glucocorticoid response elements (GREs) present in the *FKBP5* promoter and distal intronic regions, thus forming an ultra-short feedback loop [4]. Interestingly, a single nucleotide polymorphism (SNP) in *FKBP5*, specifically the T “risk allele” of the C/T SNP rs1360780 located in intron 2, has previously been demonstrated to enhance *FKBP5* transcription by moderating GR-induced epigenetic changes at multiple intronic GREs, including those within intron 7 [5].

Recently, increased DNA methylation of loci within *GR* and *FKBP5* has been identified as potential markers of HPA axis dysfunction [6, 7]. DNA methylation involves the covalent addition of a methyl group to carbon C5 of cytosine dinucleotides to create 5-methylcytosine (5mC) [8]. This can result in an altered chromatin state and elicit long-term changes in gene expression and pathological dysfunctions [8]. Importantly, unlike genetic mutations, DNA methylation is reversible and therapies targeting DNA methyltransferases have been successfully applied to the management of certain neoplastic diseases [9]. Understanding the role of DNA methylation modifications in HPA axis regulation may lead to unique opportunities for preventive and therapeutic interventions aimed at restoring HPA axis function in metabolic diseases.

White adipose tissue (WAT), the primary site for energy storage in mammals, is distributed throughout the body in several depots which differ in structural organization, cellular function and associated risk for developing metabolic disease [10–12]. While abdominal obesity is more closely associated with metabolic disease due to its greater contribution to the systemic free fatty acid pool and chronic low-grade inflammation, accumulation of lower body (i.e. gluteal) fat is associated with a reduced incidence of type 2 diabetes (T2D) and cardiovascular disease (CVD), possibly owing to reduced lipolytic potential and greater differentiation capacity of adipocytes within this depot [13]. While the mechanisms underlying these depot differences in metabolic risk remain elusive, it has been speculated that intrinsic variations in gene expression and epigenetic signatures between adipose depots may be responsible [11, 13, 14].

There is accumulating evidence to suggest that disruption of genes within the HPA axis may play a causal role in obesity and associated metabolic diseases [15–27]. However, to the best of our knowledge, no studies have examined the potential pathologic epigenetic modulation of HPA axis-regulatory genes in adipose tissue, a highly active endocrine organ and primary energy reservoir in humans. The aim of the current study was therefore to determine [1] the DNA methylation profiles of *GR* and *FKBP5* in gluteal (GSAT) and abdominal (ASAT) WAT from obese and normal weight South African women, and [2] the association of these methylation profiles with markers of adiposity, insulin resistance and inflammatory status. We propose that hypermethylation of *GR* and *FKBP5*, key HPA axis-regulatory genes, is associated with metabolic dysfunction and that assessing methylation patterns of *GR* and *FKBP5* in GSAT and ASAT depots may provide a better understanding of the intrinsic differences between abdominal and lower body obesity.

## Results

### Participant characteristics

The characteristics of participants in this study have been described in detail previously [28] and are summarized in Table 1. The participants were all female, 51.85% black and 48.15% white South African. The obese women were significantly older than normal weight women (*p* = 0.004). There were no significant differences in socioeconomic score (SES), current smoking status or weekly alcohol consumption between obese and normal weight women.

**Table 1:** Participant characteristics

	Normal weight (n=27)	Obese (n=27)	***P*** value
**DEMOGRAPHIC AND LIFESTYLE FACTORS**			
**Age (years)**	24 (22-26)	28 (24-36)	0.004
**Ethnicity**			
**Black**^a^	14 (51.9)	14 (51.9)	
**SES**	16.8 (11.6-22.6)	20.1 (10.6-23.1)	0.843
**Smoking (yes)**^a^	7 (25.9)	7 (25.9)	0.936
**Alcohol consumption (g)**	4.7 (0-24.6)	3.8 (0-16.1)	0.854
**BODY COMPOSITION**			
**BMI (kg/m**^**2**^**)**	22.9 (21.7-23.7)	38.3 (34.3-41.6)	<0.001
**Fat (%)**	29.7 (25.9-35.1)	47.0 (43.5-49.6)	<0.001
**BODY FAT DISTRIBUTION**			
**Waist circumference (cm)**	78.3 (74.0-82.0)	112.5 (102.6-118.5)	<0.001
**GSAT (kg)**	4.6 (4.0-5.2)	5.9 (5.4-6.5)	0.001
**ASAT (cm**^**2**^**)**	169.7 (134.7-225.6)	558.2 (480.2-620.3)	<0.001
**VAT (cm**^**2**^**)**	54.3 (42.6-77.2)	107.7 (80.3-173.7)	<0.001
**METABOLIC PARAMETERS**			
**Fasting Glucose (mmol/L)**	4.4 (4.1-4.6)	4.5 (4.4-4. 7)	0.027
**Fasting Insulin (mU/L)**	5.1 (3.5-8.5)	12.5 (7.1-16.9)	0.001
**HOMA-IR**	0.9 (0.7-1.7)	2.5 (1.1-3.3)	<0.001
**S**_**I**_ **(·10**^**-4**^ **min**^**-1**^**/[μU/ml])**	4.1 (1.4-6.2)	1.8 (1.0-3.6)	0.011
**C-reactive protein (mg/l)**	1.2 (0.6-3.8)	7.9 (3.4-8.7)	<0.001
**Adiponectin (ng/ml)**	6.5 (4.1-9.5)	3.5 (2.1-5.8)	<0.001
**Leptin (ng/ml)**	15.2 (7.8-20.5)	59.0 (47.9-66.2)	<0.001

Data expressed as the median (25th–75th percentile) or as ^a^count (percentage)

Abbreviations: *BMI* body mass index, *GSAT* gluteal subcutaneous adipose tissue, *HOMA-IR* Homeostatic model of insulin resistance, *SAT* subcutaneous adipose tissue, *SES* socioeconomic score, *S*_I_ insulin sensitivity index, *VAT* visceral adipose tissue

By design, all measures of body composition and fat distribution were greater in obese compared to normal weight women. While no differences in fasting glucose levels were observed between the groups, the obese women displayed higher systemic inflammation (as measured by c-reactive protein), hyperinsulinemia and insulin resistance (as measured by homeostasis model for insulin resistance (HOMA-IR), increased leptin and reduced adiponectin concentrations) and reduced insulin sensitivity (*S*_I_) compared to normal weight women.

### GR and FKBP5 methylation analyses

**Fig. 1:**
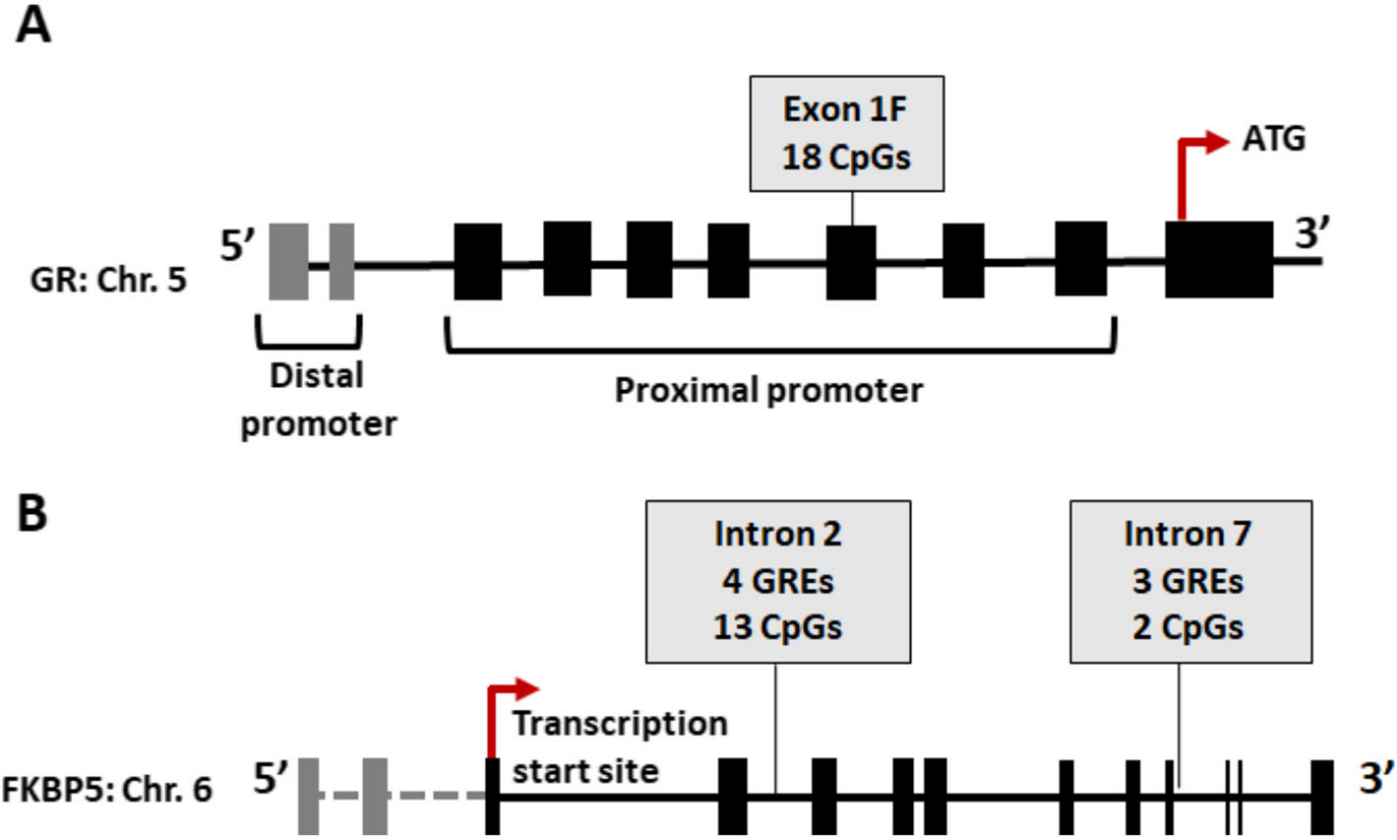
Schematic diagram of the **a** *GR* and **b** *FKBP5* genes, illustrating the CpG sites analysed in the proximal 1F promoter and introns 2 and 7, respectively. The transcription start sites and glucocorticoid response elements (GREs) within *FKBP5* are indicated

We used pyrosequencing to analyse 18 CpG sites situated within exon 1F of the proximal *GR* promoter (Fig. 1a), as well as 13 CpG sites within intron 2 and two CpG sites within intron 7 of *FKBP5* (Fig. 1b). Methylation of *GR* and *FKBP5* were analysed in GSAT and ASAT biopsies as representatives of lower body and central obesity, respectively.

**Fig. 2:**
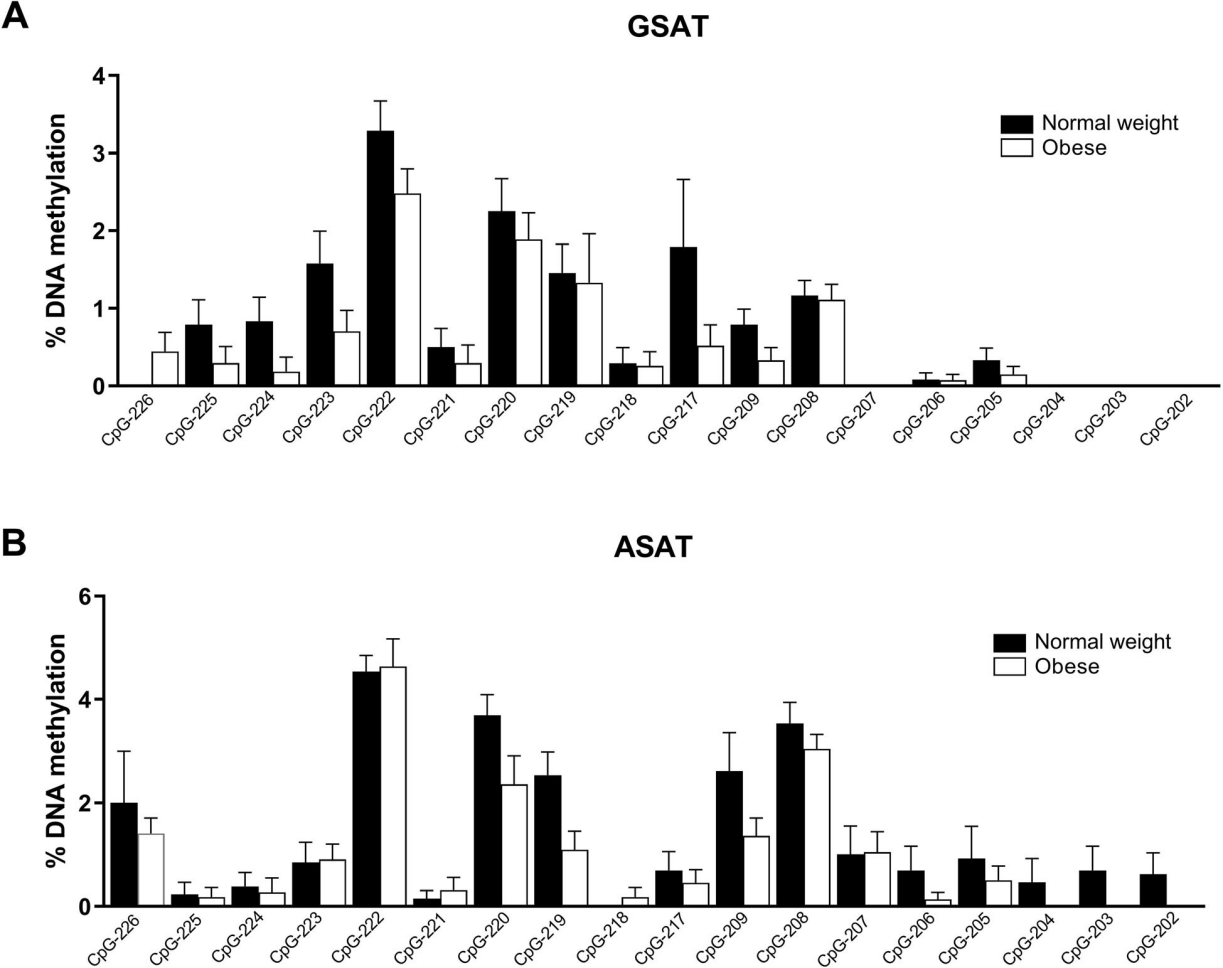
Pyrosequencing analysis of *GR* methylation in gluteal (GSAT) and abdominal (ASAT) adipose tissue. Percentage methylation of individual CpG sites in GSAT (**a**) and ASAT (**b**) of normal weight and obese South African women. Data represented as mean ± SD. (GSAT, *n* = 27 per group; ASAT, *n* = 13-22 per group)

Results revealed low *GR* methylation levels in both GSAT (Fig. 2a) and ASAT (Fig. 2b) depots, as well as a large degree of inter-individual variation at all CpG sites, irrespective of obesity. In GSAT, *GR* methylation appeared to be lower in obese women compared to normal weight women, although these differences were not significant (Fig. 2a). No consistent differences in DNA methylation patterns in ASAT were observed between obese compared to normal weight women (Fig. 2b).

**Fig. 3:**
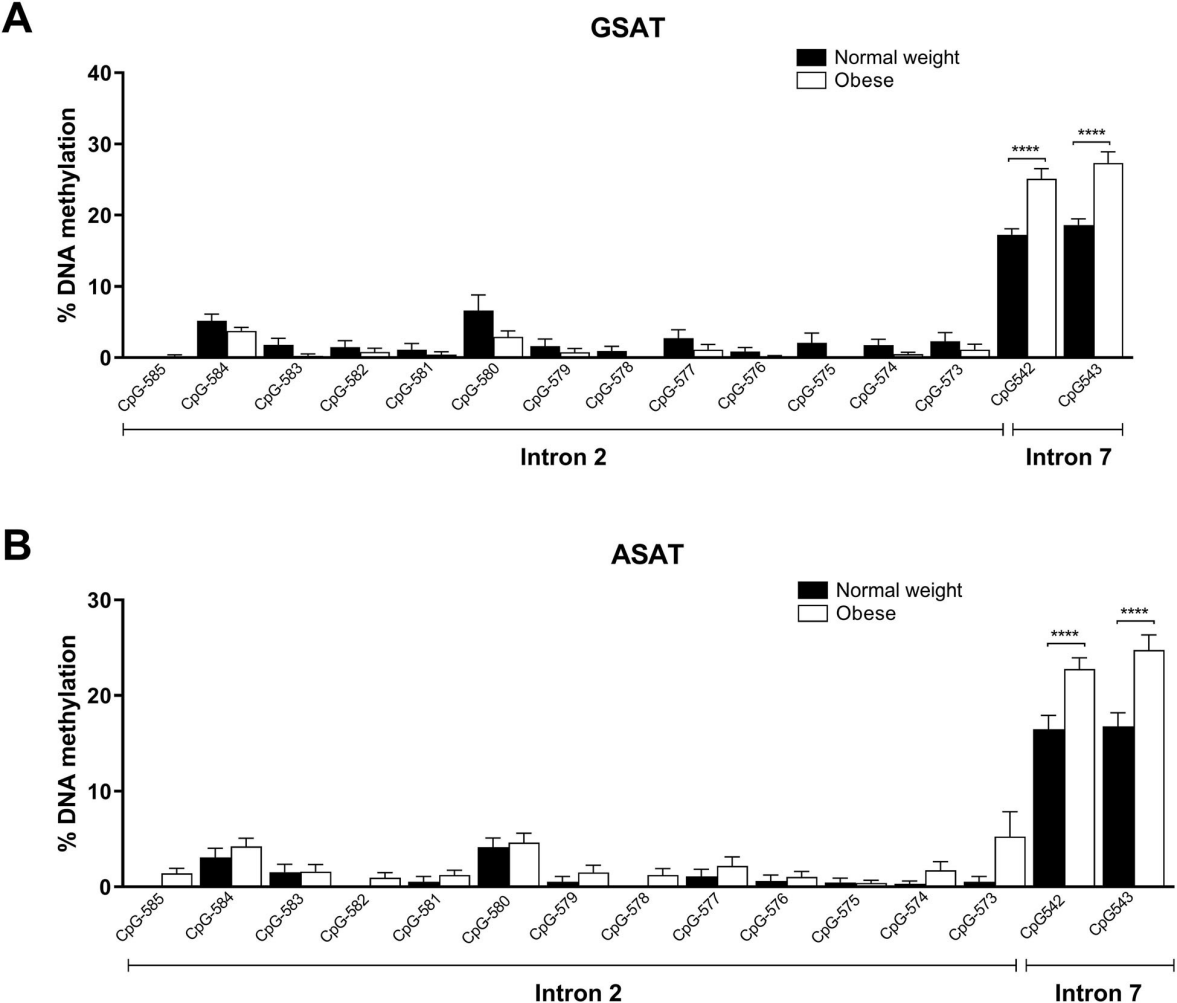
Pyrosequencing analysis of *FKBP5* methylation in gluteal (GSAT) and abdominal (ASAT) adipose tissue. Percentage methylation of individual CpG sites in GSAT (**a**) and ASAT (**b**) of normal weight and obese South African women. Data represented as mean ± SD. (GSAT, *n* = 27 per group; ASAT, *n* = 13–22 per group). Statistical significance analysed using OPLS-DA. ****p < 0.0001

An analysis of *FKBP5* intron 2 methylation levels in GSAT and ASAT revealed no significant differences between obese compared to normal weight women (Fig. 3a, b). For *FKBP5* intron 7, methylation levels of CpG542 (137,847 bp from TSS) and CpG543 (137,872 bp from TSS) were approximately 10% higher in obese compared to normal weight women (GSAT, *p* < 0.001; ASAT, *p* < 0.001), which was consistent for both depots (Fig. 3a, b).

### FKBP5 methylation associations with obesity and parameters of metabolic function

**Fig. 4:**
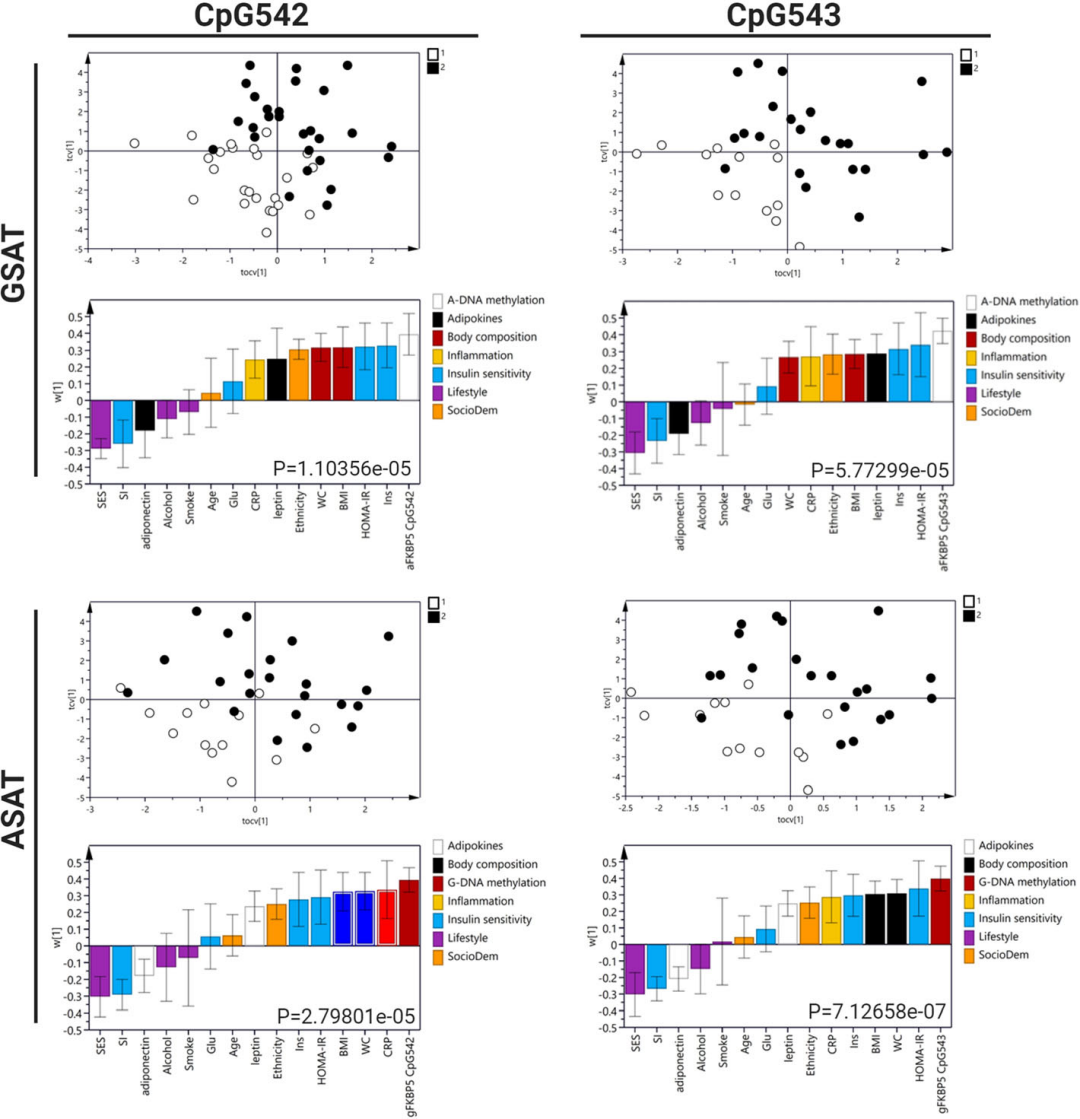
Multivariate analysis of adiposity (BMI and waist circumference (WC)), markers of insulin sensitivity (HOMA-IR, fasting insulin), plasma adipokines (increased leptin and reduced adiponectin), systemic inflammation (c-reactive protein), sociodemographic factors [age, ethnicity, socioeconomic status (SES), alcohol consumption and smoking status] and DNA methylation of *FKBP5* CpG542 and CpG543 in GSAT and ASAT from obese and normal weight women (OPLS-DA, CV-ANOVA: *p* < 0.0001). Top panels: OPLS-DA cross-validated scores that describe participant variability. Lower panels: the body composition, sociodemographic, insulin sensitivity, inflammation, adipokines and *FKBP5* methylation (OPLS weights, w [1] with 95% CI) that discriminate between obese [2] and normal weight [1] women

The differential methylation patterns observed in obese versus normal weight women prompted us to further explore the associations of these CpG sites with clinical and biochemical features of obesity and metabolic dysfunction. We explored DNA methylation associations using orthogonal partial least-squares discriminate analysis (OPLS-DA) (Fig. 4). The OPLS score plot for each model revealed a separation between the obese and normal weight women (Fig. 4, top panels). The loading plots (Fig. 4, lower panels), which signify the basis of the score plot clustering, revealed that CpG542 and CpG543 were highly correlated between ASAT and GSAT (CpG542: rs = 0.698, *p* < 0.001; CpG543: rs = 0.765, *p* < 0.001), and positively associated with measures of adiposity, including BMI and waist circumference (WC) in both adipose depots. Similarly, positive associations were observed for markers of insulin resistance (HOMA-IR, fasting insulin, reduced insulin sensitivity, increased leptin and reduced adiponectin) and systemic inflammation (c-reactive protein). CpG542 and CpG543 methylation also significantly associated with ethnicity and SES in both depots. Additionally, CpG543 methylation associated with alcohol consumption in ASAT. Consequently, all associations were adjusted accordingly for significant covariates. When the adjustments were made, all associations were maintained; however, the associations with insulin sensitivity lost statistical significance (Table S1).

### GR and FKBP5 mRNA expression levels in SAT

**Fig. 5:**
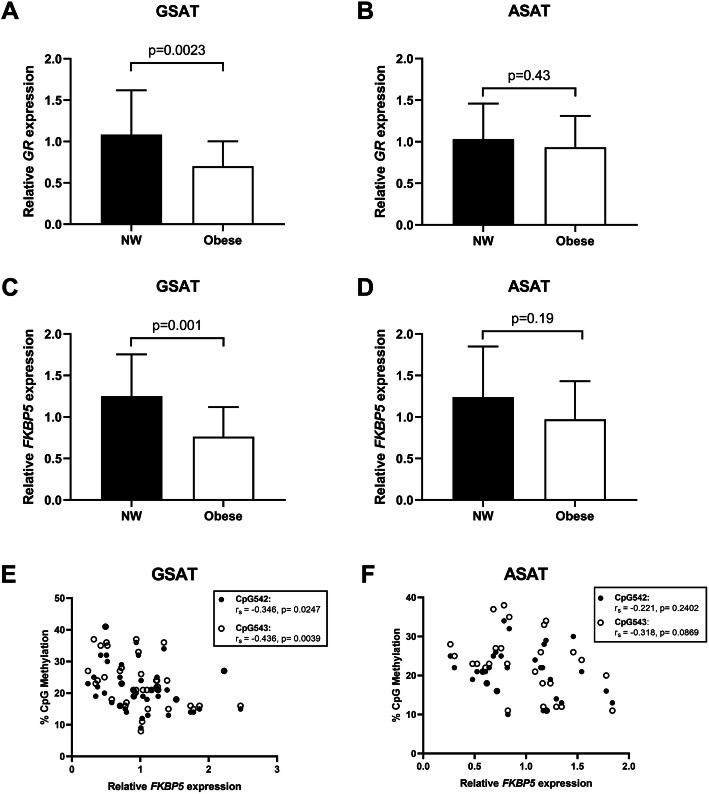
*GR* and *FKBP5* mRNA expression in gluteal (GSAT) and abdominal (ASAT) adipose tissue. Relative mRNA expression of *GR* in GSAT (**a**) and ASAT (**b**) and *FKBP5* in GSAT (**c**) or ASAT (**d**) from normal weight (NW) and obese South African women. Data represent mean ± SD (GSAT, *n* = 27 per group; ASAT, *n* = 13–22 per group). Spearman’s correlation analysis of *FKBP5* CpG542 and CPG543 methylation and *FKBP5* mRNA levels in GSAT (**e**) and ASAT (**f**) from normal weight and obese black and white South African women (*n* = 27 per group). Linear regression lines used for descriptive purposes only

To examine whether altered CpG methylation corresponded with changes in *GR* and *FKBP5* expression, we measured their messenger ribonucleic acid (mRNA) status in GSAT and ASAT using quantitative real-time PCR (qRT-PCR). GSAT from obese women had ~ 30% lower GR mRNA expression levels (*p* < 0.010) compared to normal weight women (Fig. 5a), while no differences were observed in ASAT (Fig. 5b). Similarly, *FKBP5* mRNA expression levels in GSAT were reduced by ~ 40% in obese compared to normal weight women (Fig. 5c) but was unchanged in ASAT (Fig. 5d). Correlation analyses revealed a significant inverse relationship between *FKBP5* mRNA levels and CpG542/3 methylation in GSAT, but not in ASAT (Fig. 5e, f). Furthermore, *FKBP5* mRNA negatively associated with markers of adiposity, insulin resistance and systemic inflammation in GSAT, but not in ASAT (Table S2).

### Effect of rs1360780 SNP on FKBP5 methylation and gene expression

In order to assess the potential moderating role of the rs1360780 SNP on *FKBP5* methylation and gene expression data in our study, we genotyped all participants’ SAT samples for this SNP. Using the combined sample of obese and normal weight participants, we determined the genotype frequencies to be 0.44 (*n* = 21), 0.38 (*n* = 19) and 0.16 (*n* = 6) for the CC, CT and TT (rare homozygous “risk allele”) genotypes, respectively, and we observed no significant deviations from the Hardy-Weinberg equilibrium in this sample population (*p* = 0.12) (data not shown).

**Fig. 6:**
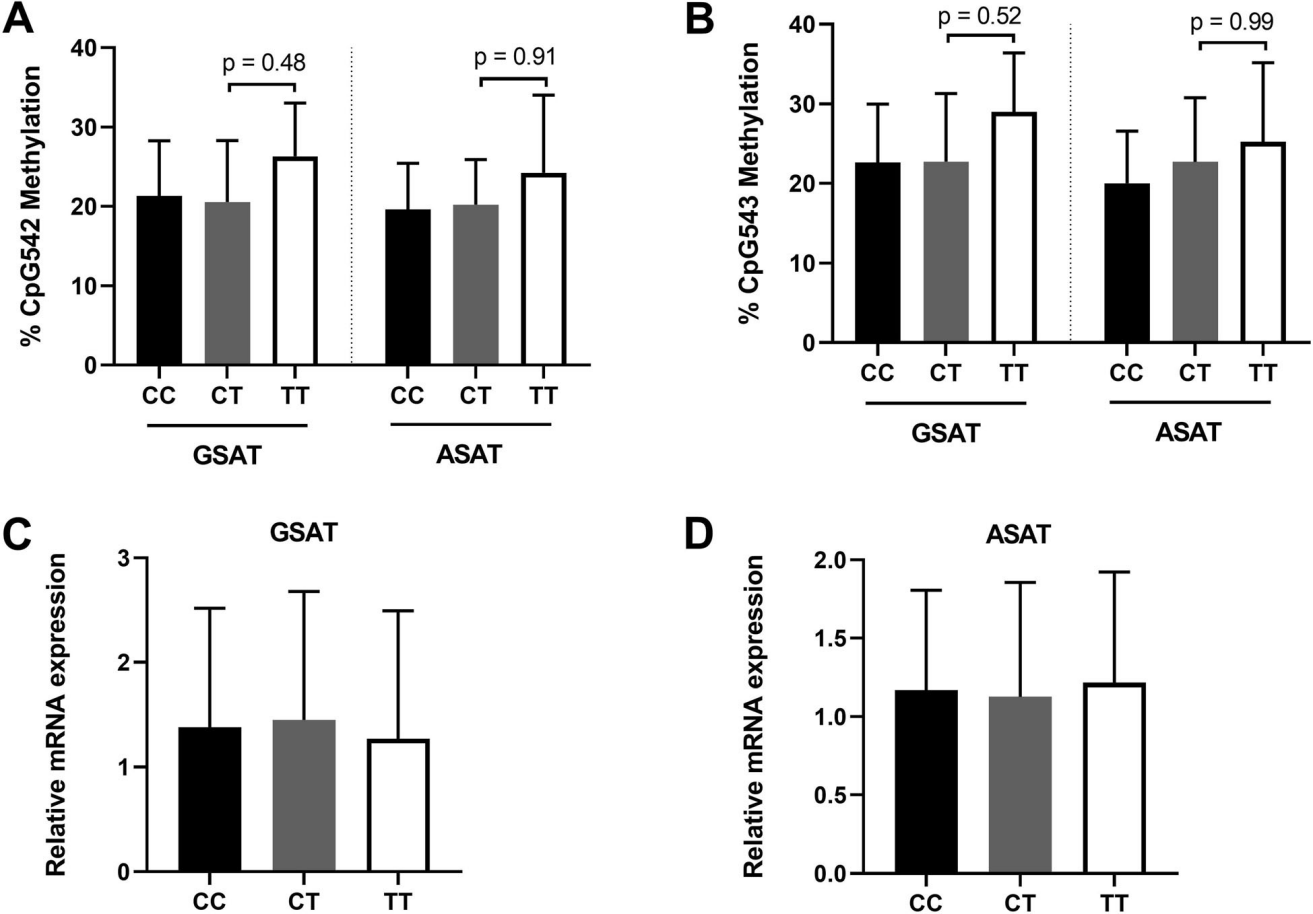
Genotype effect of rs1360780 on *FKBP5* CpG542 (**a**) and CpG543 (**b**) methylation and relative mRNA expression levels in gluteal (GSAT) (**c**) and abdominal (ASAT) (**d**) adipose tissue from normal weight and obese South African women. Data represent mean ± SD

We next stratified the samples based on genotype and observed no significant differences in *FKBP5* methylation at CpG543 (Fig. 6a) or CpG543 (Fig. 6b) between the CC, CT and TT genotypes in both GSAT and ASAT depots. Similarly, no differences were observed in *FKBP5* mRNA expression between any of the genotypes in GSAT (Fig. 6c) or ASAT (Fig. 6d). Taken together, these data demonstrate that *FKBP5* methylation and gene expression levels in obese and normal weight women in our study were not influenced by the rs1360780 SNP.

## Discussion

Obese individuals have a higher risk of developing T2D and CVD; however, these diseases are often diagnosed years after the onset of insulin and glucose dysregulation, during which micro- and macrovascular complications can occur. It is thus crucial to understand the early pathogenic mechanisms of obesity and insulin resistance sequelae in order to develop early preventative interventions. Our present data provide novel evidence that DNA methylation of two intronic CpG sites in *FKBP5*, a key player of the HPA axis and stress response, is altered in SAT from obese compared to normal weight women. Moreover, multivariable regression analysis of these CpG sites revealed a positive relationship with measures of adiposity (BMI and waist circumference), insulin resistance (HOMA-IR, fasting insulin, increased leptin and reduced adiponectin levels) and systemic inflammation (CRP), all of which are established risk factors for T2D and CVD.

Our analysis of *GR* methylation within exon 1F revealed considerable inter-individual variability in our sample population. This is consistent with a previous study assessing methylation of the same *GR* promoter region in patients with subclinical atherosclerosis [27] and also agrees with other epigenetic studies in complex diseases [29]. While the magnitude of *GR* methylation alterations between obese and normal weight women are less pronounced compared to those identified in, for example, various types of malignant disorders [30, 31], they are consistent with genome-wide association studies showing subtle changes in *GR* methylation, including sites within exon 1F, in numerous disorders [32–34]. Furthermore, *GR* mRNA expression was downregulated in GSAT from our obese study population [19], and although its expression did not correlate with methylation levels (data not shown), it is now widely accepted that DNA methylation marks are functionally complex and may orchestrate other important regulatory events, including alternative splicing and even the promotion of gene transcription [8]. However, we cannot rule out the possibility of other genetic/epigenetic mechanisms underlying *GR* regulation in this study, such as the presence of SNPs or inhibition by non-coding RNAs and/or histone modifications [22, 35].

While *FKBP5* has been extensively studied in the context of neuropsychiatric diseases [5, 36–39], several recent studies have uncovered a metabolic role for this gene [40]. Indeed, preclinical studies using animal models have demonstrated that deletion of the *FKBP5* gene protects mice from diet-induced obesity and hepatic steatosis, and pharmacological inhibition of *FKBP5* improves their metabolic health [41, 42]. In humans, genetic variants and hypomethylation within intron 2 of *FKBP5* have been associated with Cushing’s syndrome [24]. *FKBP5* SNPs have also been linked to insulin resistance and T2D traits [23, 25] and reduced weight loss following bariatric surgery [43]. More recently, hypermethylation of two CpG sites in *FKBP5* intron 2 in peripheral blood were associated with metabolic risk and cardiovascular disease traits, including BMI and WC, in individuals with T2D [44]. While this study was limited by a small sample size, it agrees with our findings on the association between higher intronic *FKBP5* methylation and an increased risk for obesity and insulin resistance.

In agreement with the longstanding association of DNA methylation with gene silencing, we observed that CpG542 and CpG543 hypermethylation in obese women inversely correlated with *FKBP5* transcript levels in GSAT. Although these CpGs do not reside within the proximal *FKBP5* promoter, it is now appreciated that many steroid-regulated genes lack response elements in their promoters and are rather regulated through distal binding sites [45]. Furthermore, while it was beyond the scope of this study to elucidate the transcription factors that occupy and regulate this region, we note that CpG542 and CpG543 are located either directly within, or flank, biologically validated consensus glucocorticoid response elements (GREs), which have been shown to come into direct contact with the *FKBP5* transcriptional start site and RNA polymerase II, via three-dimensional chromatin loops, and influence *FKBP5* transcription [39]. In agreement with this are several reports demonstrating that exposure of human peripheral blood cells to dexamethasone, a GR agonist, can induce rapid demethylation and transcription of *FKBP5* via these GREs [5, 46]. Furthermore, while previous studies have shown that *FKBP5* demethylation and mRNA induction by GR is influenced by the rs1360780 SNP in human peripheral blood from both African American and Caucasian populations [5, 46–48], this was not observed in our study, albeit in a limited sample size. These findings may suggest that *FKBP5* regulation is tissue-specific; however, confirmation of this in larger, more representative studies is required.

Obesity is characterized by a state of chronic, low-grade inflammation, which may be a key mechanism underlying the development of several obesity-associated diseases including T2D and CVD. Higher levels of inflammatory cytokines have been shown to cause a downregulation of GR expression and/or functionality of the receptor, resulting in glucocorticoid resistance, a condition characterized by insensitivity of tissues to the actions of glucocorticoids [49]. In adipose tissue, the development of glucocorticoid resistance by tumour necrosis factor α (TNFα) treatment was also shown to inhibit GR function by promoting the expression of GRβ, a nuclear localized GR isoform which acts as a dominant negative of GRα transcriptional activity [50]. Interestingly, the obese women in our study had a more proinflammatory adipose tissue profile [51], including elevated expression levels of TNFα and migration inhibitory factor cytokines, as well as the CCL2 chemokine and macrophage markers, CD68 and CD163, which was accompanied by reduced GRα mRNA compared to normal weight women [51]. This observation corroborates with other studies demonstrating a negative association between GRα mRNA levels and measures of inflammation, obesity and insulin resistance [17, 49, 52].

**Fig. 7:**
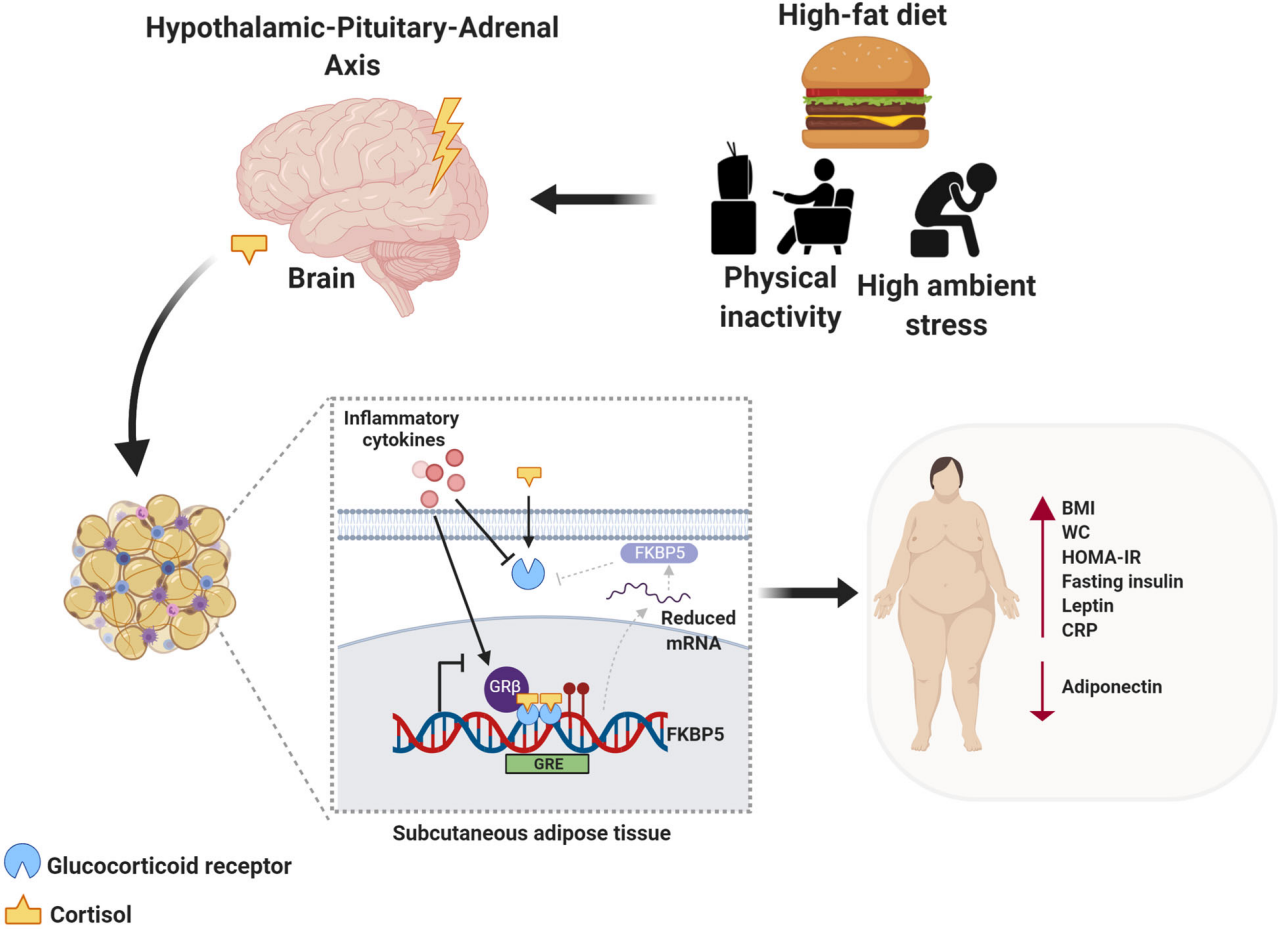
Schematic proposed model of study findings showing how metabolic stressors lead to obesity and inflammation-induced downregulation of GR, possibly through glucocorticoid resistance and induction of the non-ligand binding GRβ isoform, and consequently, hypermethylation of *FKBP5* at selected CpGs (CpG542 and CpG543) located within intron 7 in subcutaneous adipose tissue. Reduced *FKBP5* mRNA expression would result in a loss of the ultra-short negative feedback loop between FKBP5 and GR (grey arrows), thus altering the biologic response to glucocorticoids. DNA methylation alterations in *FKBP5* CpG542/3 are associated with increased clinical risk factors for disease, including adiposity (high BMI, WC and leptin), inflammation (high CRP) and insulin resistance (elevated fasting insulin and HOMA-IR, low adiponectin) and reduced insulin sensitivity. This figure was created using BioRender (https://biorender.com/)

Based on this, we propose a model whereby obesity- and inflammation-induced downregulation of *GR*, and possible induction of GRβ, may augment *FKBP5* methylation and, consequently, reduce *FKBP5* transcription in GSAT (Fig. 7). These events, in turn, result in a loss of the ultra-short negative feedback loop between FKBP5 and GR, thus altering the biologic response to glucocorticoids. The change in glucocorticoid sensitivity in GSAT, triggered by low GR, may represent a beneficial feedback mechanism protecting adipocytes from dysregulated HPA axis activity during the progression of obesity and insulin resistance. It is unclear why *GR* and *FKBP5* mRNA levels were not similarly reduced in ASAT. It is possible that statistical significance was not obtained due to the use of a smaller sample size available for this tissue type. Alternatively, the effects on GR signaling may be dampened in this depot due to ethnic differences in inflammatory profiles of SAT between black and white women in our study population. Indeed, a previous study using the same sample population showed that GSAT from black women had higher expression of inflammatory cytokines, macrophage markers and leptin compared to ASAT [52].

Our study has several strengths. To our knowledge, this is the first study to assess both *GR* and *FKBP5* DNA methylation in the same population of obese and normal weight individuals, providing a more comprehensive understanding of the interplay between these genes. Moreover, in contrast to most DNA methylation studies in obesity, we assessed *GR* and *FKBP5* methylation levels in adipose tissue rather than peripheral blood. Adipose tissue is the primary affected organ during the development of obesity, and to date, few DNA methylation studies have been performed using this tissue type. The comparisons that were made between gluteal and abdominal SAT further strengthen this study, as they enabled the exploration of unique features that may contribute to the variability in associated metabolic risk between these depots. Indeed, notable differences in the magnitude of metabolic risk associations (as determined by multivariable regression β-coefficients) were observed for CpG542 in ASAT and GSAT, whereas the β-coefficients for CpG543 associations were relatively similar between depots. This observation raises the possibility that the role of CpG543 methylation is more highly conserved between the different adipose depots compared to CpG542, although additional experiments are required to confirm this.

Several limitations should also be noted when interpreting the results of this study. Firstly, adipose tissue contains a vascular-stromal fraction in which macrophages, fibroblasts, endothelial cells and preadipocytes reside, and we therefore cannot exclude the possible contribution of these cell types to the DNA methylation profiles obtained for *GR* and *FKBP5* in our study. While we acknowledge that many studies using whole blood, for example, control for cellular heterogeneity by measuring DNA methylation in individual cell types following cell sorting or adjusting for direct measured cell counts, we were unable to do this in our study due to limited availability of tissue. Furthermore, whereas the use of post hoc regression models to adjust for blood cell mixture is facilitated by the availability of reference datasets on cell types in blood, to the best of our knowledge, such reference datasets are not available for adipose tissue and also may not be representative of our study population [53]. Another study limitation is the use of only female participants from South Africa, and therefore, the generalizability to other race/ethnic groups, or males, is unknown. Furthermore, as a result of our cross-sectional study design, the causal relationship between methylation changes and obesity or metabolic traits cannot be determined, and these warrant further investigation in larger, longitudinal study cohorts. While pyrosequencing is a highly sensitive and reliable method for DNA methylation analysis, it only allows for the investigation of small genomic regions [54]. It is therefore possible that our analysis does not reflect the methylation effect of other genomic regions of *GR* and *FKBP5*. In particular, *FKBP5* contains numerous transcriptional regulatory regions which are distributed throughout the gene, including the promoter and other intronic enhancer regions which harbour functional GREs [5, 46] as well as CCCTC-binding factor (CTCF) sites [46]. Our analysis of the effect of the rs1360780 SNP on *FKBP5* methylation and gene expression levels should also be interpreted in the context of a small sample size, and larger, more representative studies will be required to confirm this finding. Lastly, the molecular function of FKBP5 in adipose tissue during the progression of obesity remains to be elucidated. FKBP5 knockout adipocyte cell culture models may shed light on this and forms part of future studies.

## Conclusions

Our study findings provide the first demonstration of an association between *FKBP5* methylation in intron 7 in SAT and established markers of obesity and metabolic risk [55, 56]. While these findings are purely correlative and cannot be used to determine causality, they add to a growing list of studies marking aberrant DNA methylation of HPA axis genes as clinically meaningful risk factors for metabolic diseases [57]. Future studies using longitudinal cohorts should shed light on whether *FKBP5* hypermethylation is a cause or consequence of metabolic dysregulation. Moreover, it remains to be determined whether altered *FKBP5* methylation patterns in SAT are reflected in blood, which could be used to assess potential risk of developing metabolic disease. Given the long time-course of T2D development and the reversible nature of epigenetic changes, our results demonstrate the significance of the HPA axis in metabolic dysfunction and mark constituents of this pathway as potential targets for preventative and therapeutic interventions for obesity and its complications.

## Methods

### Study population

The study was approved by the Human Research Ethics Committee of the Faculty of Health Sciences of the University of Cape Town. The study sample consisted of 27 obese (body mass index (BMI) ≥ 30 kg/m^2^) and 27 normal weight (BMI < 25 kg/m^2^) premenopausal, urban-dwelling South African women who were recruited as previously described [28]. All participants were of self-reported European or Black African (Xhosa) ancestry (both parents), aged 18–45 years without any known diseases, were not pregnant or lactating and were not taking medication for diabetes, hypertension, human immunodeficiency virus/acquired immunodeficiency syndrome (HIV/AIDS) or other metabolic-related disorders. Informed, written consent was obtained from each participant prior to testing.

### Participants characteristics

#### Assessment of demographic and lifestyle factors

Baseline information was obtained from participants using standardized questionnaires as previously described [28]. Briefly, questionnaire information included participant demographics, age, race, current smoking status, level of alcohol consumption (estimated using a food frequency questionnaire) and information on asset index, education level, household crowding and employment status which were used to calculate a socioeconomic score (SES) as previously described [51].

#### Body composition

Assessment of body composition was described in detail previously [28]. Basic anthropometric measurements including height, weight and waist circumference were recorded and BMI was calculated as weight (kg)/height^2^ (m^2^). Total fat mass and gluteal subcutaneous adipose was measured by dual-energy X-ray absorptiometry (DXA; Discovery-W, Software version 12.6; Hologic Inc., Bedford, MA, USA) while visceral and abdominal tissue mass was measured by computerized tomography (CT; Toshiba X-press Helical Scanner, Tokyo, Japan) [28].

#### Biochemical and metabolic analysis

After an overnight fast, blood samples were drawn for the measurement of plasma glucose and serum insulin concentrations, which were used to estimate insulin resistance, based on the homeostasis model of insulin resistance (HOMA-IR) [58]. Participants underwent an insulin-modified frequently sampled intravenous glucose tolerance test (FSIGT) as previously described [59], from which the insulin sensitivity index (*S*_I_) was measured using Bergman’s minimal model of glucose kinetics [60].

#### Fat biopsies

Adipose tissue biopsies were obtained from superficial abdominal subcutaneous adipose tissue (ASAT) and gluteal subcutaneous adipose tissue (GSAT) depots using a mini liposuction method as previously described [51]. The tissues were placed in vials, snap frozen in liquid nitrogen and stored at − 80^o^C for subsequent gene expression and DNA methylation analysis.

### DNA extraction

Genomic DNA was extracted from GSAT and ASAT using the QIAamp DNA kit (Qiagen. Valencia, CA, USA) according to the manufacturer’s instructions. DNA quality and concentrations were assessed using the NanoDrop ND-1000 Spectrophotometer (NanoDrop Products, Wilmington, USA) and Qubit fluorometer (Life Technologies, CA, USA), respectively.

### Bisulfite conversion

Treatment of DNA with sodium bisulfite converts all unmethylated cytosine bases into uracil, allowing for the identification of methylated, unconverted cytosines by pyrosequencing [61]. Bisulfite conversion of genomic DNA from adipose tissue biopsies were performed using the EpiTect DNA kit (Qiagen, Hilden, Germany), according to the manufacturer’s instructions. The following PCR conditions were used for the conversion: 95 °C for 5 min, 60 °C for 10 min, 95 °C for 5 min, 60 °C for 10 min and 4 °C for less than 20 h. Bisulphite-treated DNA were desulphonated, washed and eluted prior to use in PCR and pyrosequencing experiments.

### Pyrosequencing

Percent DNA methylation of *GR* and *FKBP5* was determined by pyrosequencing. The *GR* gene has nine alternative first exons that are controlled independently by separate promoters [15]. Exon 1F, situated within a 3-kb CpG island, is the most widely studied, and differential methylation of CpGs within this region has been previously associated with subclinical atherosclerosis [27]. Thus, for the current study, pyrosequencing assays were designed to this region. For *FKBP5*, we assessed CpG sites within introns 2 and 7 which span experimentally validated GREs and have previously been implicated in metabolic disorders [15, 24, 44]. All pyrosequencing assays were designed by EpigenDX (Worcester, MA, USA). PCR of 25 ng bisulfite converted DNA was carried out using the PyroMark PCR Kit (Qiagen, Valencia, CA, USA) under the following PCR conditions: *GR* (EpigenDX assay ADS1342): 95 °C for 15 min; 45 × (95 °C for 30 s; 56 °C for 30 s; 72 °C for 30 s); 72 °C for 5 min; *GR* (EpigenDX assay ADS2386): 95 °C for 15 min; 45 × (95 °C for 30 s; 54 °C for 30 s; 72 °C for 30 s); 72 °C for 5 min; *FKBP5* (EpigenDX assay ADS3269): 95 °C for 15 min; 45 × (95 °C for 30 s; 60 °C for 30 s; 68 °C for 30 s); 68 °C for 5 min; *FKBP5* (EpigenDX assay ADS3828): 95 °C for 15 min; 45 × (95 °C for 30 s; 56 °C for 30 s; 68 °C for 30 s); 68 °C for 5 min. PCR reactions were carried out in a Veriti 96-well Thermal Cycler (Thermo Fisher, Waltham, MA, USA). The quality of PCR amplicons was assessed by agarose gel electrophoresis and amplicons were stored at 4 °C until pyrosequencing. Single-stranded amplicons were annealed to pyrosequencing primers (EpigenDX, Worcester, MA, USA) and subjected to primer extension and nucleotide incorporation using the PyroMark Q96 MD pyrosequencer and PyroMark Gold Q95 reagents (Qiagen, Valencia, CA, USA) according to the manufacturer’s instructions. Sequencing analysis was performed using the PyroMark Q96 software (Version 1.0.10; Qiagen) program, which determined percent DNA methylation at all CpG dinucleotides downstream of the annealed primer at > 90% precision. All pyrosequencing assays were validated using different ratios of methylated:unmethylated bisulfite converted DNA (0, 10, 25, 50, 75, 90 and 100%) (Qiagen, Valencia, CA, USA), from which standard curves were constructed to determine primer sensitivity (Fig. S1). Pyrosequencing assay validation also included quality controls containing either of the following: sequencing primer and annealing buffer only; biotinylated PCR primer and annealing buffer only; sequencing primer, biotinylated PCR primer and annealing buffer only; PCR product and annealing buffer only; PCR no template control, sequencing primer and annealing buffer only. Each pyrosequencing run contained no template negative controls, and bisulfite conversion controls were incorporated within each assay sequence to assess conversion efficiency. Assays were repeated if any of the inbuilt quality control measures were flagged. The nomenclature of *GR* and *FKBP5* CpGs are based on their sequencing identities from EpigenDX (Worcester, MA, USA).

### RNA extraction, reverse transcription and real-time PCR

Total RNA was extracted from GSAT and ASAT biopsies using the RNeasy/miRNeasy mini Kit (Qiagen, CA, USA) according to the manufacturer’s instructions. RNA quantity and purity were determined using the NanoDrop ND-1000 Spectrophotometer (NanoDrop Products, DE, USA). Complementary DNA (cDNA) was synthesized using the High-Capacity cDNA Reverse Transcription Kit (Applied Biosystems, CA, USA), and mRNA levels of FKBP5, GR and reference genes 18S, PPIA and RPLP0 quantified using TaqMan Universal PCR Master Mix and TaqMan gene expression assays (Applied Biosystems, CA, USA), according to the manufacturer’s instructions. Relative expression levels were calculated using the standard curve method. Normfinder, a mathematical model of gene expression [62], was used to confirm the stable expression of the reference genes and identified RPLP0 as the best normalization gene. Thus, expression levels were normalized to the RPLP0 endogenous control.

#### FKBP5 SNP genotyping

The FKBP5 rs1360780 (C/T) polymorphisms were genotyped using qRT-PCR with Taqman genotyping assays (Applied Biosystems, Massachusetts, USA) on the QuantStudio™ 7 Flex Real-Time PCR System (Applied Biosystems, Massachusetts, USA). Briefly, qRT-PCR was performed using 9.4 ng of DNA, 5 μl of Taqman Master Mix and 0.25 μl of 40× TaqMan SNP genotyping assay in a total volume of 10 μl, according to the manufacturer’s instructions. The following PCR conditions were used: 10 min at 95 C (initial denaturation/enzyme activation), 15 s at 95 C (denaturation) and 60 s at 60 C (annealing/extension) for 40 cycles. For quality control, eight samples were randomly selected and genotyped in duplicate. Positive and negative controls were included on all plates.

### Statistical analysis

Data are presented as the mean and standard deviation (SD) if normally distributed or the median and interquartile range (25th and 75th percentile) if not normally distributed. The Shapiro-Wilk test was used to test for normality. Student’s *t* tests (normal distribution) and Mann-Whitney tests (non-normal distribution) were used to compare anthropometric, metabolic and gene expression differences between obese and normal weight women. In order to explore site-specific DNA methylation differences between obese and normal weight women, as well as to examine associations with metabolic parameters, multivariable analysis was used. This analysis helps to avoid multiple comparisons and has the ability to deal with correlated variables. Firstly, the complete dataset was inspected using principal component analysis (PCA) to detect groupings and outliers. We then performed orthogonal partial least-squares discriminate analysis (OPLS-DA) to examine differences in site-specific DNA methylation between obese and normal weight women. The CpG sites that were differentially methylated between obese and normal weight women were then used in OPLS analysis to explore associations with measures of insulin sensitivity, body composition, plasma adipokines, systemic inflammation, sociodemographic and lifestyle factors. The models were validated based on analysis of variance (ANOVA) of the cross-validated OPLS scores (CV-ANOVA) for significance testing [63]. Variables were considered significant when fulfilling the statistical significance criteria using post hoc linear regression on loadings calculated from the validated OPLS models using a 95% confidence level [64]. Multivariable linear regression was then conducted to explore the associations between the DNA methylation sites and the metabolic outcomes, adjusting for significant confounding factors, shown in the OPLS models. The correlation between differentially methylated CpG sites and mRNA levels, and association of mRNA levels with metabolic parameters, were assessed using Spearman’s correlation analysis. Since mRNA levels did not correlate with lifestyle and sociodemographic variables, no post hoc adjustments were made for these data. Genotype-stratified DNA methylation and gene expression data were compared between obese and normal weight women using the non-parametric Kruskal-Wallis test. Hardy-Weinberg equilibrium of the genotyped *FKBP5* SNP (rs1360780) was tested using a chi-square goodness of fit test. Data were analysed using STATA version 14.0 (StataCorp, College station, TX, USA), GraphPad Prism (v8.2.1) and SIMCA v. 15.02. Significance was accepted as *p* < 0.05.

## Supplementary information

**Additional file 1:** **Figure S1**. Sensitivity of pyrosequencing assays used to interrogate *GR* and *FKBP5*. Standard curves for *GR* CpG-226 to CpG-217 (A), GR CpG-209 to CpG-202 (B), FKBP5 CpG -585 to CpG-573 (C) and *FKBP5* CpG542 and CpG543 (D). **Table S1**. Correlation between DNA methylation and cardiometabolic risk factors. Data expressed as the β-coefficient (p-value) adjusted for ethnicity, socioeconomic status and *alcohol consumption. Abbreviations: ASAT, abdominal subcutaneous adipose tissue; BMI, body mass index; CRP, c-reactive protein; GSAT, gluteal subcutaneous adipose tissue; HOMA-IR, Homeostatic model assessment-insulin resistance; *S*_I_, insulin sensitivity index; WC, waist circumference. **Table S2**. Correlation between *FKBP5* mRNA levels and cardiometabolic risk factors. Data expressed as the Spearman’s r-coefficient (*p*-value). Abbreviations: ASAT, abdominal subcutaneous adipose tissue; BMI, body mass index; CRP, c-reactive protein; GSAT, gluteal subcutaneous adipose tissue; HOMA-IR, Homeostatic model assessment-insulin resistance; SES, socioeconomic status, *S*_I_, insulin sensitivity index; WC, waist circumference.

## Data Availability

The datasets used and/or analysed during the current study are available from the corresponding author on reasonable request.

## Abbreviations

ASAT: Abdominal subcutaneous adipose tissue
AUC: Area under the curve
BMI: Body mass index
CRP: C-reactive protein
CVD: Cardiovascular disease
FKBP5: FK506 binding protein 5
GR: Glucocorticoid receptor
GSAT: Gluteal subcutaneous adipose tissue
HOMA-IR: Homeostatic model of insulin resistance
OPLS: Orthogonal partial least-squares
OPLS-DA: Orthogonal partial least-squares discriminate analysis
PCA: Principle component analysis
PCR: Polymerase chain reaction
qRT-PCR: Quantitative real-time PCR
SAT: Subcutaneous adipose tissue
SES: Socioeconomic score
*S*_I_: Insulin sensitivity index
TSS: Transcriptional start site
T2D: Type 2 diabetes
VAT: Visceral adipose tissue
WC: Waist circumference
