# Supplementary material for: DNA methylation of FKBP5 in South African women: associations with obesity and insulin resistance
Source: Clin Epigenetics. 2020 Sep 21;12:141. doi: 10.1186/s13148-020-00932-3 (PMC7507280; doi:10.1186/s13148-020-00932-3)
Supplement: Supplementary file 1 — Additional file 1: Figure S1. Sensitivity of pyrosequencing assays used to interrogate GR and FKBP5. Standard curves for GR CpG-226 to CpG-217 (A), GR CpG-209 to CpG-202 (B), FKBP5 CpG -585 to CpG-573 (C) and FKBP5 CpG542 and CpG543 (D). Table S1. Correlation between DNA methylation and cardiometabolic risk factors. Data expressed as the β-coefficient (p-value) adjusted for ethnicity, socioeconomic status and *alcohol consumption. Abbreviations: ASAT, abdominal subcutaneous adipose tissue; BMI, body mass index; CRP, c-reactive protein; GSAT, gluteal subcutaneous adipose tissue; HOMA-IR, Homeostatic model assessment-insulin resistance; SI, insulin sensitivity index; WC, waist circumference. Table S2. Correlation between FKBP5 mRNA levels and cardiometabolic risk factors. Data expressed as the Spearman’s r-coefficient (p-value). Abbreviations: ASAT, abdominal subcutaneous adipose tissue; BMI, body mass index; CRP, c-reactive protein; GSAT, gluteal subcutaneous adipose tissue; HOMA-IR, Homeostatic model assessment-insulin resistance; SES, socioeconomic status, SI, insulin sensitivity index; WC, waist circumference. [file 13148_2020_932_MOESM1_ESM.zip › table S1.docx]

|  | **ASAT** | | **GSAT** | |
| --- | --- | --- | --- | --- |
|  | **CpG542** | **CpG543** | **CpG542** | **CpG543** |
| **ADIPOSITY** |  | | | |
| **BMI (kg/m^2^)** | 0. 0009 (<0.001) | 0.0008 (<0.001)* | 0.0008 (<0.001) | 0.0003 (<0.001) |
| **WC (cm)** | 0.0001 (<0.001) | 0.0001 (<0.001)* | 0.0000 (<0.001) | 0.0001 (<0.001) |
| **INSULIN RESISTANCE/SENSITIVITY** | | | | |
| **Fasting glucose (mmol/L)** | 465.1860 (0.273) | -83.9944 (0.119)* | -29.02256 (0.097) | -73.2407 (0.084) |
| **Fasting insulin (pmol/L)** | 24.9131 (0.006) | 3.6833 (0.003)* | 1.0351 (0.010) | 2.4639 (0.011) |
| **HOMA-IR** | 8.4195 (0.030) | 1.3225 (0.010)* | 0.04341 (0.012) | 1.0110 (0.016) |
| **S_i_ (x10^-4^min^-1^/(μUmL^-1^))** | -0.7119 (0.123) | -0.1109 (0.066)* | -0.0229 (0.303) | -0.0422 (0.432) |
| **CIRCULATING INFLAMMATORY MARKERS** | | | | |
| **Adiponectin (ng/ml)** | -3.8548 (0.005) | -0.6003 (0.001)* | -0.1667 (0.009) | -0.4742 (0.002) |
| **Leptin (ng/ml)** | 2.1478 (0.032) | 0.3129 (0.010)* | 0.1584 (<0.001) | 0. 4364 (<0.001) |
| **CRP (mg/l)** | 0.8973 (<0.001) | 0.1026 (<0.001)* | 0. 0359 (0.001) | 0.0998 (<0.001) |

Data expressed as the β-coefficient (p-value) adjusted for ethnicity, socioeconomic status and *alcohol consumption.

Abbreviations: ASAT, abdominal subcutaneous adipose tissue; BMI, body mass index; CRP, c-reactive protein; GSAT, gluteal subcutaneous adipose tissue; HOMA-IR, Homeostatic model assessment-insulin resistance; SI, insulin sensitivity index; WC, waist circumference.
