# Supplementary material for: DNA methylation of FKBP5 in South African women: associations with obesity and insulin resistance
Source: Clin Epigenetics. 2020 Sep 21;12:141. doi: 10.1186/s13148-020-00932-3 (PMC7507280; doi:10.1186/s13148-020-00932-3)
Supplement: Supplementary file 1 — Additional file 1: Figure S1. Sensitivity of pyrosequencing assays used to interrogate GR and FKBP5. Standard curves for GR CpG-226 to CpG-217 (A), GR CpG-209 to CpG-202 (B), FKBP5 CpG -585 to CpG-573 (C) and FKBP5 CpG542 and CpG543 (D). Table S1. Correlation between DNA methylation and cardiometabolic risk factors. Data expressed as the β-coefficient (p-value) adjusted for ethnicity, socioeconomic status and *alcohol consumption. Abbreviations: ASAT, abdominal subcutaneous adipose tissue; BMI, body mass index; CRP, c-reactive protein; GSAT, gluteal subcutaneous adipose tissue; HOMA-IR, Homeostatic model assessment-insulin resistance; SI, insulin sensitivity index; WC, waist circumference. Table S2. Correlation between FKBP5 mRNA levels and cardiometabolic risk factors. Data expressed as the Spearman’s r-coefficient (p-value). Abbreviations: ASAT, abdominal subcutaneous adipose tissue; BMI, body mass index; CRP, c-reactive protein; GSAT, gluteal subcutaneous adipose tissue; HOMA-IR, Homeostatic model assessment-insulin resistance; SES, socioeconomic status, SI, insulin sensitivity index; WC, waist circumference. [file 13148_2020_932_MOESM1_ESM.zip › table S2.docx]

|  | **ASAT** | **GSAT** |
| --- | --- | --- |
| **DEMOGRAPHIC AND LIFESTYLE FACTORS** | | |
| **Age (years)** | -0.0713 (0.689) | -0.0864 (0.560) |
| **Ethnicity** | -0.1049 (0.555) | -0.2410 (0.099) |
| **SES** | 0.0483 (0.786) | 0.1987 (0.176) |
| **Smoking (yes)^†^** | -0.1148 (0.525) | -0.0491 (0.743) |
| **Alcohol consumption (g)** | 0.1494 (0.415) | 0.2042 (0.169) |
| **ADIPOSITY** | | |
| **BMI (kg/m^2^)** | 0.0298 (0.867) | -0.2931 (0.043) |
| **WC (cm)** | -0.0546 (0.759) | -0.3338 (0.020) |
| **INSULIN RESISTANCE/SENSITIVITY** | | |
| **Fasting glucose (mmol/L)** |  |  |
| **Fasting insulin (pmol/L)** | -0.0286 (0.873) | -0.3585 (0.012) |
| **HOMA-IR** | -0.1163 (0.513) | -0.3821 (0.007) |
| **S_i_ (x10^-4^min^-1^/(μUmL^-1^))** | -0.0907 (0.621) | 0.2987 (0.044) |
| **CIRCULATING INFLAMMATORY MARKERS** | | |
| **Adiponectin (ng/ml)** | -0.0188 (0.916) | 0.3118 (0.031) |
| **Leptin (ng/ml)** | -0.0634 (0.730) | -0.4265 (0.005) |
| **CRP (mg/l)** | -0.1200 (0.499) | -0.4751 (<0.001) |

Data expressed as the Spearman’s r-coefficient (p-value).

Abbreviations: ASAT, abdominal subcutaneous adipose tissue; BMI, body mass index; CRP, c-reactive protein; GSAT, gluteal subcutaneous adipose tissue; HOMA-IR, Homeostatic model assessment-insulin resistance; SES, socioeconomic status, SI, insulin sensitivity index; WC, waist circumference.
