# Supplementary figures and images for: DNA methylation of FKBP5 in South African women: associations with obesity and insulin resistance
Source: Clin Epigenetics. 2020 Sep 21;12:141. doi: 10.1186/s13148-020-00932-3 (PMC7507280; doi:10.1186/s13148-020-00932-3)

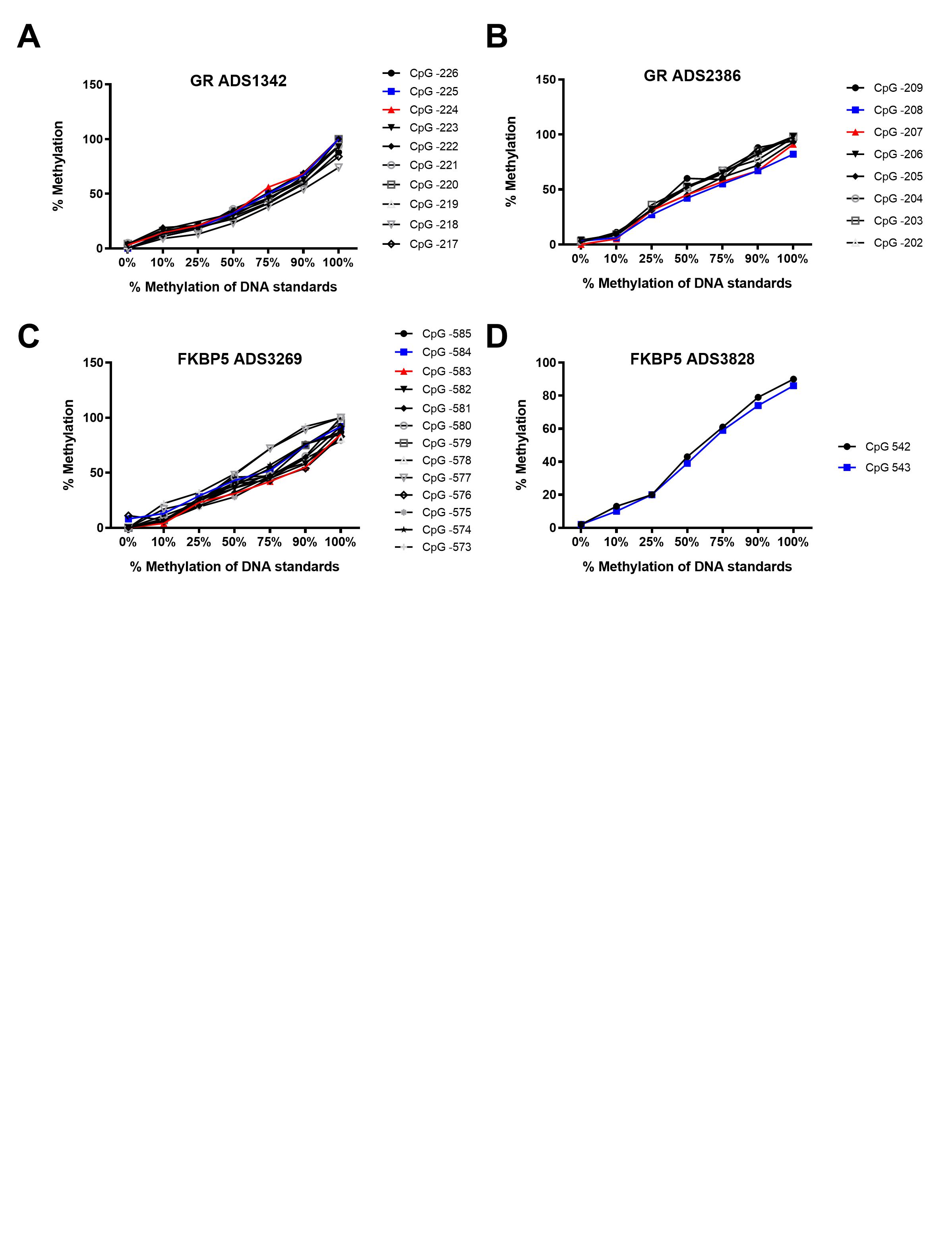

Supplement: Supplementary file 1 — Additional file 1: Figure S1. Sensitivity of pyrosequencing assays used to interrogate GR and FKBP5. Standard curves for GR CpG-226 to CpG-217 (A), GR CpG-209 to CpG-202 (B), FKBP5 CpG -585 to CpG-573 (C) and FKBP5 CpG542 and CpG543 (D). Table S1. Correlation between DNA methylation and cardiometabolic risk factors. Data expressed as the β-coefficient (p-value) adjusted for ethnicity, socioeconomic status and *alcohol consumption. Abbreviations: ASAT, abdominal subcutaneous adipose tissue; BMI, body mass index; CRP, c-reactive protein; GSAT, gluteal subcutaneous adipose tissue; HOMA-IR, Homeostatic model assessment-insulin resistance; SI, insulin sensitivity index; WC, waist circumference. Table S2. Correlation between FKBP5 mRNA levels and cardiometabolic risk factors. Data expressed as the Spearman’s r-coefficient (p-value). Abbreviations: ASAT, abdominal subcutaneous adipose tissue; BMI, body mass index; CRP, c-reactive protein; GSAT, gluteal subcutaneous adipose tissue; HOMA-IR, Homeostatic model assessment-insulin resistance; SES, socioeconomic status, SI, insulin sensitivity index; WC, waist circumference. [file 13148_2020_932_MOESM1_ESM.zip › Figure S1.jpg]
